# The renoprotective effect of esaxerenone independent of blood pressure lowering: a post hoc mediation analysis of the ESAX-DN trial

**DOI:** 10.1038/s41440-022-01008-w

**Published:** 2022-09-13

**Authors:** Yasuyuki Okuda, Sadayoshi Ito, Naoki Kashihara, Kenichi Shikata, Masaomi Nangaku, Takashi Wada, Tomoko Sawanobori, Masataka Taguri

**Affiliations:** 1 Data Intelligence Department, Daiichi Sankyo Co., Ltd, Tokyo, Japan; 2 Division of Nephrology, Endocrinology and Vascular Medicine, Department of Medicine, Tohoku University School of Medicine, Sendai, Japan; 3 Katta General Hospital, Shiroishi, Japan; 4 Department of Nephrology and Hypertension, Kawasaki Medical School, Kurashiki, Japan; 5 Center for Innovative Clinical Medicine, Okayama University Hospital, Okayama, Japan; 6 Division of Nephrology and Endocrinology, Graduate School of Medicine, The University of Tokyo, Tokyo, Japan; 7 Department of Nephrology and Laboratory Medicine, Kanazawa University, Kanazawa, Japan; 8 Clinical Development Department I, Daiichi Sankyo Co., Ltd, Tokyo, Japan; 9 Department of Health Data Science, Tokyo Medical University, Tokyo, Japan

**Keywords:** Blood pressure, Diabetic nephropathy, Esaxerenone, Hypertension, Mediation analysis

## Abstract

Angiotensin-converting enzyme (ACE) inhibitors or angiotensin II receptor blockers (ARBs) are recommended as first-line drugs for hypertension with diabetic nephropathy owing to their renoprotective effect; however, their effect beyond lowering blood pressure (BP) has not been confirmed. Recent studies have shown that aldosterone plays a key role in causing renal injury; therefore, it is likely that mineralocorticoid receptor (MR) blockers inhibit aldosterone-induced renal damage in different ways from ACE inhibitors and ARBs. Therefore, we investigated the mechanism of the effect of an MR blocker on reducing the urinary albumin-to-creatinine ratio (UACR) using data from a randomized, double-blind, placebo-controlled phase 3 study (ESAX-DN) of a new nonsteroidal MR blocker, esaxerenone. This *post hoc* analysis used a novel statistical method to quantitatively estimate the effect of esaxerenone on UACR reduction mediated, or not mediated, by changes in systolic BP (SBP) and/or estimated glomerular filtration rate (eGFR). The proportion of the mediated effect by SBP changes to the total effect on UACR reduction was 9.8–10.7%; the UACR was reduced to 0.903–0.911 times the baseline at the end of treatment through the SBP-related pathway and to 0.422–0.426 times the baseline through the non-SBP-related pathway. Even considering both SBP and eGFR simultaneously, the proportion of the mediated effect was 21.9–28.1%. These results confirm that esaxerenone has a direct UACR-lowering effect independent of BP lowering and that its magnitude is much larger than that of the BP-dependent effect. Thus, esaxerenone could be a UACR-reducing treatment option for patients with diabetic nephropathy.

## Abstract (web summary)

This *post hoc* mediation analysis confirmed that most of the UACR-lowering effect of esaxerenone is induced by a pathway independent of BP lowering, even considering eGFR changes.

## Graphical abstract

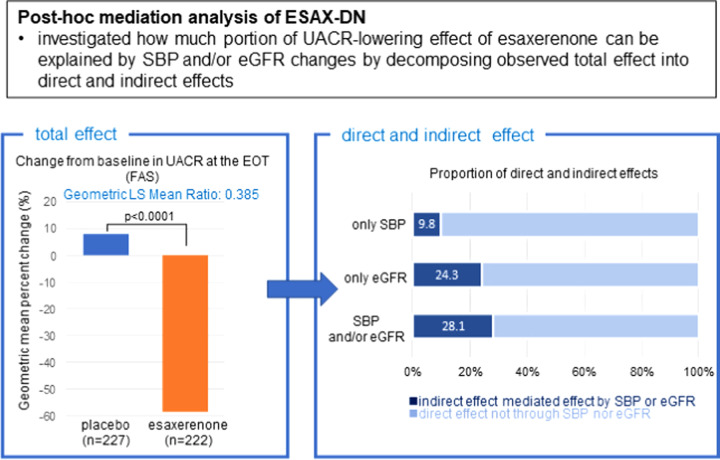

## Introduction

Renin-angiotensin system (RAS) inhibitors, such as angiotensin-converting enzyme (ACE) inhibitors or angiotensin II receptor blockers (ARBs), are recommended as first-line drugs for hypertensive patients with diabetic nephropathy mainly for their blood pressure (BP)-lowering effects [1]. However, clinical trial data have yet to confirm their renal protective effect beyond lowering BP [2–5]. Recent studies have shown that aldosterone plays a major pathologic role in causing renal injury [6]; therefore, it is likely that mineralocorticoid receptor (MR) blockers prevent aldosterone-induced renal cell damage in different ways from ACE inhibitors and ARBs [7]. If the unique effect of an MR blocker could be demonstrated, it would help identify new treatment options for hypertension associated with diabetic nephropathy.

Esaxerenone is a new nonsteroidal MR blocker that has been approved in Japan for the treatment of hypertension and is now under development by Daiichi Sankyo Co., Ltd. for diabetic nephropathy [8]. It has been demonstrated that esaxerenone has renoprotective effects in preclinical and clinical studies [9–13]. In addition, it has been suggested that esaxerenone exerts renoprotective effects independently of its antihypertensive effect in type 2 diabetic mice [14]. A multicenter, randomized, double-blind, placebo-controlled phase 3 trial (ESAX-DN) of esaxerenone confirmed that adding esaxerenone to existing RAS inhibitor therapy significantly reduced the urinary albumin-to-creatinine ratio (UACR) by 61.6% compared with a placebo (geometric least squares mean ratio: 0.384; 95% confidence interval [CI]: 0.334, 0.443) over 52 weeks of treatment in Japanese patients with type 2 diabetes and microalbuminuria [15]. In addition, in another phase 3 study, the UACR decreased by 54.6% (95% CI: 46.9, 61.3) on average from baseline (544.1 mg/g creatinine) to the end of treatment (246.8 mg/g creatinine), and improvement in microalbuminuria was shown in 51.8% of Japanese patients with type 2 diabetes and overt albuminuria during 28 weeks of treatment with esaxerenone [16]. These results clearly show that esaxerenone has a strong UACR-lowering effect in type 2 diabetic patients with albuminuria. Thus, it is now of clinical interest to determine how much of the UACR-lowering effect can be explained by lowering BP or other effects. In the ESAX-DN study, crude correlation analyses were conducted, but no strong correlation was observed between the change in the UACR and the change in BP at the end of treatment. This may indicate that the change in the UACR is irrelevant to the change in BP; however, it does not necessarily indicate that esaxerenone has a UACR-lowering effect independent of BP lowering. To confirm this, further analysis that focuses on the mechanism of the effect of esaxerenone on lowering the UACR is needed. If such an effect is confirmed, it would help to differentiate esaxerenone from other RAS inhibitors as a treatment for diabetic nephropathy.

In statistics, this type of analysis into the mechanism of an agent is called “mediation analysis” and has been used in medical and psychological research [17, 18]. In mediation analysis, the total effect of a treatment on an outcome can be divided into indirect and direct effects; indirect effects act through mediators of interest, whereas direct effects act through other pathways and not through mediators of interest [17–19]. Several novel methods to identify indirect and direct effects have been proposed based on the counterfactual-based framework [20, 21]. These statistical methods are practically useful for addressing clinical questions but have not yet been widely used in clinical research.

This *post hoc* analysis investigated whether esaxerenone has a UACR-lowering effect independent of BP lowering and quantitatively assessed its magnitude using data from the ESAX-DN study. In addition, this analysis aimed to determine whether esaxerenone can be a treatment option for patients with diabetic nephropathy.

## Methods

The reporting of this *post hoc* mediation analysis is in accordance with the consensus-based guidance for the reporting of mediation analyses of randomized trials and observational studies (A Guideline for Reporting Mediation Analyses; AGReMA Statement) [22].

### Study design and population

This was a *post hoc* analysis of a multicenter, randomized, double-blind, placebo-controlled phase 3 trial (JapicCTI-173695), the ESAX-DN study [15]. The study protocol of the ESAX-DN study was approved by the local institutional review board at each participating site and conducted in accordance with the principles of the Declaration of Helsinki and the ICH E6 Guideline for Good Clinical Practice (CPMP/ICH/135/95). All the participants in the ESAX-DN study provided written informed consent prior to enrollment.

In the ESAX-DN study, patients were randomized to either esaxerenone or placebo treatment for 52 weeks after a 4-week run-in period, continuing their treatment with RAS inhibitors at a constant dosage throughout the study. To minimize the risk of increasing serum potassium (K^+^) levels, esaxerenone or its placebo treatment was started at a dosage of 1.25 mg/d and then titrated to 2.5 mg/d if the serum K^+^ level of each patient was acceptable. Other details of the study population, such as the inclusion and exclusion criteria, have been previously published [15].

### Outcomes

The primary endpoint of the ESAX-DN study was the proportion of patients with UACR remission at the end of treatment, and the key secondary endpoint was the percentage change in the UACR from baseline at the end of treatment. The changes in BP and creatinine levels and the rate of transition to overt albuminuria were also assessed. Safety endpoints included adverse events, serum K^+^ levels, percentage changes in eGFR from baseline to the end of treatment and time-course changes in eGFR.

Of these outcomes, data for the percentage changes in the UACR from baseline to the end of treatment, changes in BP, and changes in eGFR were used in this *post hoc* analysis.

### Assessments

The UACR, calculated from first morning urine samples, was measured during the run-in period and every 4 weeks up to week 52 during the subsequent treatment period. BP and eGFR were monitored every 2 weeks up to week 8 and every 4 weeks from week 12 to week 52 during the treatment period. The eGFR with the modification in diet in renal disease was calculated using the formula modified by the Japanese Society of Nephrology [23]. Other details of the study visits and assessments have been previously reported [15].

### Clinical assumptions

Several mechanisms may explain the renoprotective effect of esaxerenone. First, esaxerenone primarily reduces BP, which then leads to a reduction in glomerular pressure, and it can also reduce urinary albumin excretion. Second, esaxerenone may directly affect renal function and may also affect urinary albumin excretion. In addition to these mechanisms, esaxerenone may have other effects on albuminuria suppression.

**Fig. 1:**
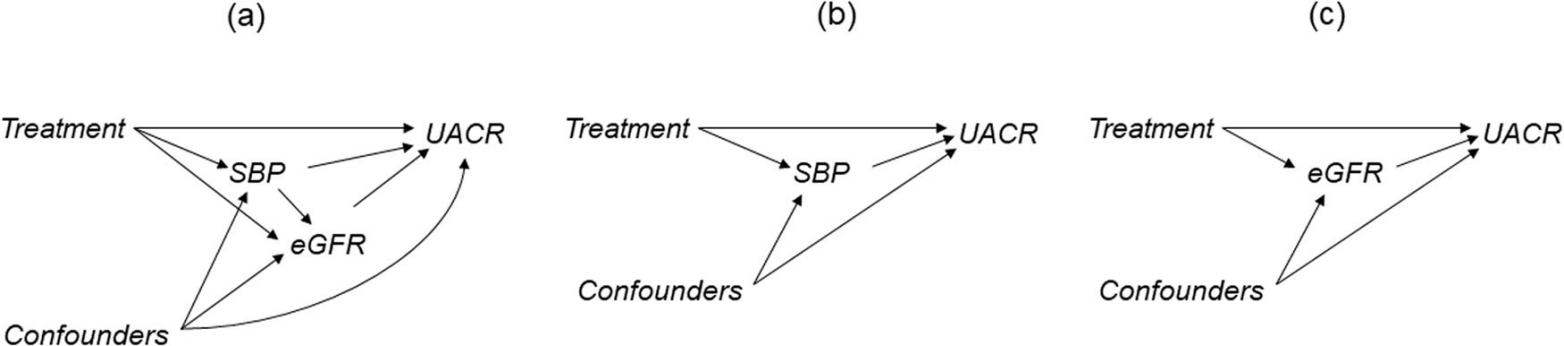
Causal relationships between variables. The graph displays the assumptions about causal relationships between variables. If an arrow points away from X and toward Y, it indicates that X causally affects Y

Based on these clinical assumptions, we considered SBP and eGFR as mediators that reflect the changes in BP and renal function and assumed the following causal relationships between the variables in this analysis (Fig. 1a). There are four possible pathways through which treatment affects UACR changes: pathways through SBP or eGFR (i.e., treatment-SBP-UACR, treatment-SBP-eGFR-UACR, treatment-eGFR-UACR) and a pathway through neither SBP nor eGFR (i.e., treatment-UACR). The former are indirect effects, and the latter the direct effect (our primary interest in this analysis). When SBP or eGFR are considered separately, the causal relationships are simplified to only two pathways: an indirect effect through SBP or eGFR and a direct effect not through SBP or eGFR (Fig. 1b, c).

The values of SBP and eGFR change longitudinally during treatment. In addition, SBP and eGFR may affect UACR changes in different manners. Therefore, to incorporate the changes in SBP and eGFR during treatment, two types of mediator variables were considered: the cumulative average value of change from baseline up to the end of treatment and the achieved value of change from baseline to just before the end of treatment. The former assumes a cumulative effect, and the latter assumes an acute effect of mediator variables on the outcome.

### Statistical analysis

To quantitatively investigate direct and indirect effects, we used the concepts of the “natural direct” effect and the “indirect effect” [20]. The natural direct effect is defined as the between-treatment comparison of the effect on the outcome if the mediator levels were set to what they would have been if the control treatment (e.g., placebo) was initiated. The natural indirect effect is defined as the comparison of the mediator effect on the outcome between mediator levels that would have been observed under the experimental or control treatment while the treatment was set to the experimental treatment (e.g., esaxerenone). The sum of natural direct and indirect effects is the total effect defined as the treatment effect comparison between when all subjects had experimental treatment and when all subjects had control treatment, the so-called intention-to-treat (ITT) effect in randomized trials. Further detailed explanations based on the causal inference framework are included in Supplementary Text 1.

Some strong assumptions are required to identify natural direct and indirect effects: (i) no unmeasured confounding of the exposure-outcome relationship; (ii) no unmeasured confounding of the outcome-mediator relationship; (iii) no unmeasured confounding of the exposure-mediator relationship; and (iv) no mediator-outcome confounder that is itself affected by the exposure. In randomized studies such as the ESAX-DN study, assumptions (i) and (iii) would hold because of the randomization of the treatment, but (ii) and (iv) would not necessarily hold and cannot be confirmed by the data.

To overcome this problem with the assumptions, a methodology with multiple mediators was considered in this analysis. The confounder for the mediator-outcome relationship was considered the mediator, and the joint indirect effect of all mediators was estimated [20]. Another advantage of this method is that we do not need to specify the causal relationships between mediators (i.e., BP and eGFR in this analysis).

To estimate the natural direct and indirect effects, we used the regression-based approach for single mediator and multiple mediator settings (details are shown in Supplementary Text 2). In the multivariate linear regression model, we included the treatment, mediator(s), interaction between treatment and mediator(s), and baseline covariates (i.e., baseline values of the UACR and mediators) as confounders.

In addition to the direct and indirect effects, the proportion of the mediated effect was calculated as (indirect effect/total effect) × 100 (%), which expresses how much of the total effect is mediated through mediators. The CIs for estimates were constructed based on the bootstrap method with 1000 replications.

As in the ESAX-DN study, the log-transformed UACR was used in the analyses and back-transformed and expressed in the original scale in the results (i.e., the effect on UACR reduction was expressed as the geometric mean ratio to baseline, compared with placebo). All statistical analyses were performed using SAS System Release 9.4 (SAS Institute Japan Ltd., Tokyo, Japan).

## Results

### Patients

A total of 455 patients were randomized, but 6 patients were excluded from the full analysis set (*n* = 449) due to missing primary endpoints. All the patients in the full analysis set were included in this *post hoc* analysis. Detailed information on patient characteristics and the time-course changes in the UACR, BP, and eGFR have been previously reported [15].

### Direct and indirect effects

The total effect, the placebo-adjusted geometric mean ratio to baseline, was 0.385, indicating that esaxerenone reduced the UACR to 0.385 times the baseline compared to placebo in total. We attempted to decompose this total effect of 0.385 into indirect and direct effects using mediation analysis.

The results are summarized in Table 1, 2. When the cumulative means of SBP and eGFR were considered separately (Table 1), the indirect effect of SBP was 0.911 (95% CI: 0.831, 0.987), and the direct effect was 0.422 (95% CI: 0.351, 0.497), indicating that the total effect of 0.385 was divided into 0.911 × 0.422. An alternative explanation is that esaxerenone reduced the UACR to 0.911 times the baseline through BP-related pathways and to 0.422 times the baseline through non-BP-related pathways at the end of treatment. The proportion of the mediated effect by SBP was 9.8% (95% CI: 2.0, 20.1). The indirect effect of eGFR was 0.793 (95% CI: 0.721, 0.864), the direct effect was 0.485 (95% CI: 0.417, 0.569), and the proportion of the mediated effect through the eGFR-related pathway was 24.3% (95% CI: 16.3, 34.8). Although the indirect effect of eGFR was slightly larger than that of SBP, the proportion of the mediated effect was not very large, even for eGFR. Both for SBP and eGFR, the results were almost consistent with respect to the type of variable (i.e., cumulative mean or achieved value): proportions of the mediated effect were 10.7% (95% CI: 4.6, 19.2) and 16.4% (95% CI: 10.6, 25.3) for the SBP-related pathway and for the eGFR-related pathway, respectively. However, there seems to be a tendency for eGFR to have a cumulative effect (i.e., 24.3% vs. 16.4%) compared to SBP (i.e., 9.8% vs. 10.7%).

**Table 1:** Summary of mediation analysis (single mediator)

Mediator variable	Summary measure	Effect	Point estimate^a^ (95% CI^b^)	PM (95% CI^b^)
SBP	Cumulative mean^c^	TE	0.385 (0.329, 0.440)	9.8 (2.0, 20.1)%
		NDE	0.422 (0.351, 0.497)	
		NIE	0.911 (0.831, 0.987)	
	Achieved value^c^	TE	0.385 (0.335, 0.445)	10.7 (4.6, 19.2)%
		NDE	0.426 (0.370, 0.504)	
		NIE	0.903 (0.834, 0.957)	
eGFR	Cumulative mean^c^	TE	0.385 (0.336, 0.450)	24.3 (16.3, 34.8)%
		NDE	0.485 (0.417, 0.569)	
		NIE	0.793 (0.721, 0.864)	
	Achieved value^c^	TE	0.385 (0.333, 0.439)	16.4 (10.6, 25.3)%
		NDE	0.450 (0.386, 0.518)	
		NIE	0.855 (0.790, 0.908)	

*TE* total effect, *NDE* natural direct effect, *NIE* natural indirect effect, *PM* proportion of the mediated effect, *CI* confidence interval, *SBP* systolic blood pressure, *eGFR* estimated glomerular filtration rate

^a^TE, NDE, and NIE were calculated based on log-transformed UACR values, and then back-transformed, and therefore, the point estimates of the effect are expressed as the geometric mean ratio to baseline

^b^The CIs are based on the bootstrap method with 1000 replications

^c^Cumulative average is the average value up to the end of treatment and achieved value is the observed value just before the end of treatment. The former assumes a cumulative effect and the later assumes an acute effect of mediator variables on the outcome

**Table 2:** Summary of mediation analysis (multiple mediators)

Mediator variable	Summary measure	Effect	Point estimate^a^ (95% CI^b^)	PM (95% CI^b^)
SBP and eGFR	Cumulative mean^c^	TE	0.385 (0.331, 0.440)	28.1 (18.2, 41.4)%
		NDE	0.503 (0.424, 0.596)	
		NIE	0.765 (0.677, 0.838)	
	Achieved value^c^	TE	0.385 (0.331, 0.440)	21.9 (14.3, 31.2)%
		NDE	0.474 (0.403, 0.548)	
		NIE	0.812 (0.745, 0.871)	

*TE* total effect, *NDE* natural direct effect, *NIE* natural indirect effect, *PM* proportion of the mediated effect, *CI* confidence interval, *SBP* systolic blood pressure, *eGFR* estimated glomerular filtration rate

^a^TE, NDE, and NIE were calculated based on log-transformed UACR values, and then back-transformed, and therefore, the point estimates of the effect are expressed as the geometric mean ratio to baseline

^b^The CIs are based on the bootstrap method with 1000 replications

^c^Cumulative average is the average value up to the end of treatment and achieved value is the observed value just before the end of treatment. The former assumes a cumulative effect and the later assumes an acute effect of mediator variables on the outcome

When SBP and eGFR were considered simultaneously (Table 2), the proportion of the mediated effect was 28.1% (95% CI: 18.2, 41.4); the indirect effect of esaxerenone on UACR reduction mediated by SBP and/or eGFR changes was 0.765 (95% CI: 0.677, 0.838), and the direct effect mediated neither by SBP nor eGFR changes was 0.503 (95% CI: 0.424, 0.596) at the end of treatment. This means that esaxerenone reduced the UACR mostly by the pathways that are relevant to neither SBP nor eGFR changes. Similar to the single mediator analysis, the results were almost the same with respect to the type of variable, although there seemed to be a trend for a cumulative effect, possibly owing to the effect of eGFR.

Even when diastolic BP (DBP) or mean arterial pressure (MAP) was considered instead of SBP, the results were almost the same; the mediated effect by DBP or MAP accounted for a small portion of the total effect (Supplementary Table 1).

**Fig. 2:**
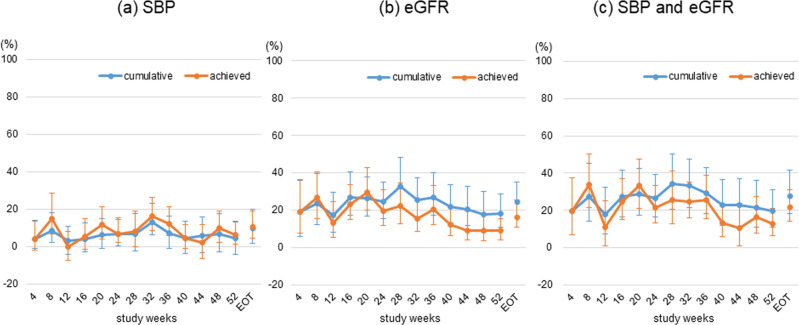
Changes in the proportion of the mediated effect by treatment duration. Changes in the proportion of the mediated effect (%) for different time points from week 4 up to week 52; SBP and eGFR separately (**a**, **b**), SBP and eGFR simultaneously (**c**). *SBP* systolic blood pressure, *eGFR* estimated glomerular filtration rate, *EOT* end of treatment

Although we consider the end of treatment as the primary time point of interest, the longitudinal effect is also worth investigating, as the ESAX-DN study showed different patterns in the longitudinal changes in the UACR, SBP, and eGFR; the UACR decreased slowly until week 24 and remained stable until the end of treatment, incremental reductions in SBP were observed up to approximately week 28 or week 32, and eGFR continued to decrease until week 24 and then remained stable in the esaxerenone group [15]. To investigate longitudinal changes in the effect of esaxerenone, we repeated the same analysis changing the endpoint from week 4 to week 52. In this supportive analysis, only patients who had a UACR value at each time point were included in the analysis; therefore, the number of patients analyzed differed slightly among time points. There was some variability in the proportion of the mediated effect; however, the results showed that most effects were consistently independent of SBP and/or eGFR changes (Fig. 2). This indicates that our findings are robust with respect to the treatment duration.

In addition, we also conducted subgroup analyses to investigate subgroup differences in the proportion of mediated effects with respect to sex (male, female), obesity (Body Mass Index < 25, 25 ≤ [kg/m^2^]) and diabetes status (HbA1c < 7.4, 7.4 ≤ [%]), baseline UACR (<100, 100 ≤ [mg/g Cr]), baseline eGFR (<60, 60 ≤ [mL/min/1.73 m^2^]), DPP4 inhibitor use (yes, no), and SGLT2 inhibitor use (yes, no). The results are almost consistent among subgroups in that the majority of the total effect was not mediated by SBP changes (Supplementary Table 2). This might support the generalizability of our findings, although there may be a sample size limitation, and the assumptions for this mediation analysis may not be valid for some subgroups.

## Discussion

In this *post hoc* analysis, we quantitatively investigated whether esaxerenone has a UACR-lowering effect independent of lowering BP, applying a novel statistical methodology to the results of a pivotal phase 3 study, ESAX-DN. To the best of our knowledge, this is the first study to use mediation analysis to investigate the direct and indirect effects of an MR blocker on lowering the UACR via, and not via, lowering BP using clinical trial data. The results show that most of the UACR-lowering effect of esaxerenone is induced by a pathway independent of BP changes, even considering eGFR changes. This suggests that esaxerenone has a UACR-lowering effect beyond lowering BP in patients with microalbuminuria.

This *post hoc* analysis statistically confirmed that esaxerenone has a direct UACR-lowering effect not mediated through lowering BP; however, the actual mechanism by which esaxerenone directly induces an antialbuminuric effect is not clear from this analysis. As discussed in a previous paper [13], sodium depletion may be one possible mechanism. In addition, preclinical studies have shown that MRs are involved in salt-induced organ damage that is independent of plasma aldosterone levels [24]. Thus, the direct effect of esaxerenone on UACR reduction may be largely due to MR blockade.

There are various discussions in the literature about microalbuminuria changes as a surrogate endpoint of diabetic kidney disease [25]. Two recent meta-analyses reported that a > 30% reduction in albuminuria confers substantial risk reduction for end-stage kidney disease [26, 27]. This *post hoc* analysis confirmed that esaxerenone has a strong direct effect on UACR reduction (57.4–57.8% relative to placebo) and therefore that the magnitude of the direct effect that is independent of BP changes can be considered clinically significant in terms of the prevention of end-stage kidney disease.

There are several methodologies that can directly handle multiple and/or time-dependent mediators [21, 28, 29]. However, such methods are often not practical, as they require additional assumptions, and their estimation is complicated. Therefore, more practical approaches using multiple and/or time-dependent mediators are desirable, as used in this analysis. Our main analysis and supportive analysis are probably sufficient for the ESAX-DN data, as the indirect mediating effect is relatively small.

The current analysis has several limitations. This was a *post hoc* analysis with no prespecified analysis plan, and the sample size of the ESAX-DN study was not designed for use in a mediation analysis. However, the sample size is probably sufficient in terms of the precision of the mediation analysis, as the CIs of the estimates were sufficiently narrow. Second, the ESAX-DN study involved only Japanese patients; thus, generalizability of the findings to other ethnic populations may be limited. In addition, the ESAX-DN data were limited to esaxerenone; therefore, we cannot determine if our findings are unique to esaxerenone or applicable to other MR blockers. Further research is needed to address these clinical questions using data from a broader population.

In conclusion, esaxerenone has a UACR-lowering effect beyond lowering BP and thus can be a treatment option for diabetic nephropathy in patients with albuminuria.

## Supplementary information

Supplementary information

## Data Availability

Deidentified individual participant data and applicable supporting clinical study documents may be available upon request at https://vivli.org/. In cases where clinical study data and supporting documents are provided pursuant to our company policies and procedures, Daiichi Sankyo Co., Ltd. will continue to protect the privacy of our clinical study participants. Details on the data sharing criteria and the procedure for requesting access are available at https://vivli.org/ourmember/daiichi-sankyo/.
