# Supplementary material for: The renoprotective effect of esaxerenone independent of blood pressure lowering: a post hoc mediation analysis of the ESAX-DN trial
Source: Hypertens Res. 2022 Sep 13;46(2):437–44. doi: 10.1038/s41440-022-01008-w (PMC9899688; doi:10.1038/s41440-022-01008-w)
Supplement: Supplementary file 1 — Supplementary information [file 41440_2022_1008_MOESM1_ESM.docx]

**Supplementary information**

**Supplementary Text 1**

We let A denote the exposure of interest, Y the outcome of interest, and M the potential mediator of interest. For example, A might denote a treatment (a: esaxerenone, or a*: placebo), Y might denote UACR changes, and M might denote SBP changes, in this study. In addition, to conduct a mediation analysis, we used the potential outcome framework. Let Y(a) and M(a) denote the outcome and mediator values, respectively, that would be observed if treatment A were set to a value a. Similarly, let Y(a,m) denote the potential outcome that would be observed if A were set to a and M were set to m. A further assumption, referred to as the consistency assumption, is often made; when A = a, the counterfactual outcomes Y(a) and M(a) are equal to the observed outcomes Y and M, respectively. Likewise, when A = a and M = m, the counterfactual outcome Y(a,M(a)) is equal to the observed outcome Y. Using the notation described above, the natural direct effect (NDE) of treatment A on outcome Y is expressed as Y(a,M(a*)) - Y(a*,M(a*)), a comparison of treatment effect while fixing the mediator M to the level M(a*). Similarly, the natural indirect effect (NIE) is a comparison of the potential outcome that would be observed when the mediator is changed from M(a*) to M(a) while fixing the treatment A to a; Y(a,M(a)) - Y(a,M(a*)). The total effect can then be decomposed into the natural direct and indirect effect as: Y(a) - Y(a*) = { Y(a,M(a*)) - Y(a*,M(a*)) } + { Y(a,M(a)) - Y(a,M(a*)) }.

**Supplementary Text 2**

First, we present a simple regression-based approach for the estimation of direct and indirect effects. Suppose assumptions (i) – (iv) are hold, that Y and M are continuous, and that the following regression models for the outcome and mediator are correctly specified using a, m and baseline covariate c:

$$E\left[ Y|a,m,c \right]＝\theta_{0}+\theta_{1}a+\theta_{2}m+\theta_{3}am+\theta_{4}c$$

$$E\left[ m|a,c \right]=\beta_{0}+\beta_{1}a+\beta_{2}c$$

Then it is shown that the natural direct effect and indirect effect are given using parameters estimated from regression models as:

$$NDE=\left\{ \theta_{1}+\theta_{3}\left( \beta_{0}+\beta_{1}a^{*}+\beta_{2}c \right) \right\}\left( a-a^{*} \right)$$

$$NIE=\left( \theta_{2}\beta_{1}+\theta_{3}\beta_{1}a \right)\left( a-a^{*} \right)$$

Second, we extend this to deal with multiple mediators. Suppose then that assumptions (i) – (iv) are hold for the vector of mediators and that the following regressions for the outcome and mediators are correctly specified and fit to the data:

$$E\left[ Y|a,m_{1},m_{2},c \right]＝\theta_{0}+\theta_{1}a+\sum_{i} \theta_{2i}m_{i}+\sum_{i} \theta_{3i}am_{i}+\theta_{4}c$$

$$E\left[ m_{i}|a,c \right]=\beta_{0i}+\beta_{1i}a+\beta_{2i}c$$

It is also shown that the controlled direct effect and natural direct and indirect effects are then given by:

$$NDE=\left\{ \theta_{1}+\sum_{i} \theta_{3i}(\beta_{0i}+\beta_{1i}a^{*}+\beta_{2i}c) \right\}\left( a-a^{*} \right)$$

$$NIE=\left( \sum_{i} \theta_{2i}\beta_{1i}+\sum_{i} \theta_{3i}\beta_{1i}a \right)\left( a-a^{*} \right)$$

We applied this method to ESAX-DN data and estimated the direct and indirect effect with 95% confidence interval using the bootstrap method.

**Supplementary Table1. Summary of** **mediation analysis (DBP and MAP)**

| **Mediator variable** | **Summary measure** | **Effect** | **Point estimate^1^ (95% CI^2^)** | **PM (95% CI^2^)** |
| --- | --- | --- | --- | --- |
| DBP | Cumulative mean^3^ | TE | 0.386 (0.332, 0.440) | 3.2 (-0.9, 9.4) % |
|  |  | NDE | 0.398 (0.341, 0.457) |  |
|  |  | NIE | 0.970 (0.915, 1.009) |  |
|  | Achieved value^3^ | TE | 0.386 (0.332, 0.440) | 6.3 (1.8, 12.9) % |
|  |  | NDE | 0.409 (0.350, 0.469) |  |
|  |  | NIE | 0.942 (0.884, 0.983) |  |
| DBP and eGFR | Cumulative mean^3^ | TE | 0.386 (0.332, 0.440) | 26.0 (18.0, 38.3) % |
|  |  | NDE | 0.494 (0.420, 0.578) |  |
|  |  | NIE | 0.781 (0.700, 0.845) |  |
|  | Achieved value^3^ | TE | 0.386 (0.332, 0.440) | 20.7 (13.8, 30.3) % |
|  |  | NDE | 0.470 (0.404, 0.544) |  |
|  |  | NIE | 0.821 (0.751, 0.845) |  |
| MAP | Cumulative mean^3^ | TE | 0.385 (0.332, 0.440) | 7.0 (0.0, 16.7) % |
|  |  | NDE | 0.411 (0.346, 0.477) |  |
|  |  | NIE | 0.935 (0.857, 1.000) |  |
|  | Achieved value^3^ | TE | 0.385 (0.332, 0.440) | 10.3 (4.1, 18.5) % |
|  |  | NDE | 0.424 (0.360, 0.490) |  |
|  |  | NIE | 0.906 (0.835, 0.960) |  |
| MAP and eGFR | Cumulative mean^3^ | TE | 0.385 (0.332, 0.440) | 27.6 (18.6, 40.7) % |
|  |  | NDE | 0.500 (0.424, 0.591) |  |
|  |  | NIE | 0.768 (0.686, 0.837) |  |
|  | Achieved value^3^ | TE | 0.385 (0.332, 0.440) | 22.7 (15.1, 32.7) % |
|  |  | NDE | 0.478 (0.408, 0.556) |  |
|  |  | NIE | 0.805 (0.737, 0.864) |  |

^1^TE, NDE, and NIE were calculated based on log-transformed UACR values, and then back-transformed, and therefore, the point estimates of the effect are expressed as the geometric mean ratio to baseline. ^2^The CIs are based on the bootstrap method with 1000 replications. ^3^Cumulative average is the average value up to the end of treatment and achieved value is the observed value just before the end of treatment. The former assumes a cumulative effect and the later assumes an acute effect of mediator variables on the outcome.

*TE* total effect, *NDE* natural direct effect, *NIE* natural indirect effect, *PM* proportion of the mediated effect, *CI* confidence interval, *DBP* diastolic blood pressure, *MAP* mean arterial pressure, *eGFR* estimated glomerular filtration rate.

**Supplementary Table2. Subgroup analysis of the proportion of the mediated effect**

|  | **SBP** | | **eGFR** | | **SBP and eGFR** | |
| --- | --- | --- | --- | --- | --- | --- |
| **Subgroup** | **Cumulative mean^1^** | **Achieved value^1^** | **Cumulative mean^1^** | **Achieved value^1^** | **Cumulative mean^1^** | **Achieved value^1^** |
| SEX |  |  |  |  |  |  |
| Male (n=345) | 7.8 (-3.5, 21.8) % | 12.2 (4.7, 22.3) % | 22.7 (12.9, 34.6) % | 16.4 (9.3, 24.7) % | 25.9 (24.7, 41.2) % | 22.3 (13.5, 33.4) % |
| Female (n=104) | 9.9 (-2.9, 28.0) % | 3.6 (-9.8, 21.5) % | 22.2 (7.4, 40.7) % | 12.1 (0.7, 28.8) % | 26.6 (8.6, 47.7) % | 14.8 (-3.2, 37.0) % |
|  |  |  |  |  |  |  |
| BMI (kg/m^2^) |  |  |  |  |  |  |
| < 25 (n=129) | 6.3 (-4.4, 22.0) % | 9.1 (0.2, 24.3) % | 20.0 (9.3, 35.3) % | 13.9 (4.3, 26.8) % | 22.5 (10.1, 42.3) % | 19.2 (7.4, 35.0) % |
| 25 ≤ (n=257) | 11.7 (0.7, 25.9) % | 11.1 (3.0, 21.9) % | 26.5 (14.7, 41.0) % | 17.1 (8.1, 28.9) % | 31.5 (17.2, 49.4) % | 22.5 (11.4, 35.2) % |
|  |  |  |  |  |  |  |
| HbA1c (%) |  |  |  |  |  |  |
| < 7.4 (n=318) | 11.3 (2.8, 23.7) % | 11.3 (2.8, 23.7) % | 25.6 (15.4, 39.4) % | 17.4 (10.1, 26.4) % | 29.6 (17.9, 44.9) % | 22.5 (13.7, 33.1) % |
| 7.4 ≤ (n=131) | -4.3 (-25.7, 16.1) % | 2.0 (-14.0, 26.3) % | 16.4 (1.6, 38.0) % | 9.3 (-3.4, 25.8) % | 12.1 (-13.9, 38.6) % | 11.5 (-7.4, 39.0) % |
|  |  |  |  |  |  |  |
| UACR (mg/g Cr) |  |  |  |  |  |  |
| < 100 (n=192) | 8.5 (-0.9, 21.4) % | 6.7 (-0.2, 16.7) % | 27.2 (13.0, 44.7) % | 22.2 (10.6, 36.7) % | 28.1 (13.4, 46.1) % | 22.9 (11.2, 37.6) % |
| 100 ≤ (n=257) | 10.0 (-2.9, 25.5) % | 14.0 (4.5, 27.2) % | 20.9 (10.3, 32.0) % | 11.4 (4.3, 19.8) % | 27.1 (11.6, 44.3) % | 21.9 (10.8, 36.2) % |
|  |  |  |  |  |  |  |
| eGFR (mL/min/1.73 m^2^) |  |  |  |  |  |  |
| < 60 (n=145) | 10.8 (-1.5, 25.8) % | 9.8 (1.2, 23.1) % | 40.1 (21.1, 64.9) % | 20.9 (6.1, 42.3) % | 41.8 (21.8, 70.0) % | 25.3 (9.2, 47.8) % |
| 60 ≤ (n=304) | 8.5 (-2.6, 22.6) % | 10.5 (2.2, 22.7) % | 22.3 (12.4, 35.0) % | 17.1 (8.5, 27.8) % | 25.3 (11.6, 40.2) % | 21.8 (11.3, 35.3) % |
|  |  |  |  |  |  |  |
| DPP4 Inhibitor use |  |  |  |  |  |  |
| Yes (n=296) | 9.3 (1.2, 19.9) % | 10.0 (2.2, 20.7) % | 21.6 (12.0, 33.4) % | 15.5 (7.5, 24.4) % | 25.2 (14.0, 39.5) % | 19.2 (9.0, 29.9) % |
| No (n=153) | 12.2 (-13.4, 36.5) % | 11.8 (2.5, 25.2) % | 30.9 (15.0, 51.2) % | 18.2 (7.1, 33.8) % | 36.9 (10.9, 65.2) % | 26.2 (12.2, 43.5) % |
|  |  |  |  |  |  |  |
| SGLT2 Inhibitor use |  |  |  |  |  |  |
| Yes (n=106) | -2.7 (-28.2, 24.9) % | -1.4 (-19.9, 30.0) % | 23.8 (5.6, 55.5) % | 13.2 (-0.8, 35.1) % | 18.2 (-10.9, 53.0) % | 9.9 (-13.2, 43.6) % |
| No (n=343) | 10.2 (1.5, 21.1) % | 11.1 (4.6, 19.9) % | 23.9 (14.7, 35.0) % | 16.5 (8.9, 24.9) % | 27.7 (16.2, 40.7) % | 21.7 (12.2, 31.6) % |
|  |  |  |  |  |  |  |

The numbers in the table assumes represent the PM (%) and CI based on the bootstrap method with 1000 replications.

^1^Cumulative average is the average value up to the end of treatment and achieved value is the observed value just before the end of treatment. The former assumes a cumulative effect and the later an acute effect of mediator variables on the outcome.

TE total effect, NDE natural direct effect, NIE natural indirect effect, PM proportion of the mediated effect, CI confidence interval, SBP systolic blood pressure, eGFR estimated glomerular filtration rate.
